# Coping and Co-creation: One Attempt and One Route to Well-Being. Part 1. Conceptual Framework

**DOI:** 10.11621/pir2021.0110

**Published:** 2021-06-30

**Authors:** Tjeerd C. Andringa, Florence C. Denham

**Affiliations:** a University of Groningen, Groningen, Netherlands; b University of Leiden, Leiden, Netherlands

**Keywords:** well-being, agency, cognition, coping, co-creation, intelligence, power, authority

## Abstract

**Background:**

All life strives to be well, but not all life is well. This suggests that cognition aimed at improving and protecting well-being might share a common core across all life forms: core cognition

**Objective:**

In this first of a two-part theoretical article, we systematically specify the evolutionary core cognition of well-being from the perspective of general living agents. In Part 2 we apply this to identity development and the theoretical approaches to well-being. This first part aims to identify the strategies and conditions for the creation and protection of generalized well-being and describes associated behavioral ontologies.

**Results:**

We defined a set of key terms that, together, specify core cognition. This set comprises quite naturally concepts like agency, behavior, need satisfaction, intelligence, authority, power, and wisdom, which are all derived from the defining properties of life. We derived coping and co-creation as two essentially different, but complementary, behavioral ontologies. Copingis for survival and targeted problem solving and aims to end the need for its activation. Co-creation is for thriving and problem prevention and aims to perpetuate its activation. Co-creation can explain the growth of the biosphere. While both strategies are essential, the successful interplay of their strengths leads to the dominance of one of them: co-creation. Absence of success leads to a dominance of coping: a coping-trap and a strong urge to curtail behavioral diversity. We summarize the key terms of core cognition and the ontologies in two tables with defined terms.

## Introduction

In this theory paper, we rigorously formulate the evolutionary roots of well-being from first principles, namely the basic demands of being and remaining alive. We identify two strategies and associated behavioral ontologies to create and protect the conditions for well-being of humans and other life-forms. Given editorial constraints and the breadth of the topic, we have separated this paper into two parts.

In Section 1 of Part 1 we derive core cognition from first principles as the necessary foundational cognition shared by all of life in the service of being (well). This section ends in a summary table of the defining terms of core cognition. The second half of Part 1 describes the opposing and complementary properties of cognition for survival (coping) and cognition for flourishing (co-creation). This section is summarized in *Table 2*, in which we oppose and contrast the key terms of both modes of cognition as separate ontologies.

Part 2 applies the developed framework. First, we shed a fresh explanatory light on the structure of identity by connecting it to coping and co-creation (in) adequacy. Second, we apply core cognition insights on a metatheoretical level. We contrast the theory of ontological security, as a near perfect example of the coping mode’s (only) route to well-being, to the “theory” of psychological safety. This is a typical example of the co-creation mode’s route to flourishing, Third, we extend the overview tables.

In the current paper we define a few dozen Core Cognition concepts **in boldface.** If these concepts pertain to core cognition in general, their definition is included in *Table 1*. If the concept, or a specific variant of it, pertains to either coping or co-creation, it is listed in *Table 2*. Together, the separate sets of concepts form behavioral ontologies for coping and co-creation. *Table 2* is organized such that concepts with complementary roles in coping and co-creation are matched. The tables and figures form a summary of this paper.

**Table 1:** Core cognition: key terms

	Core cognition key concepts with definition
Core cognition	The cognition shared by all of life.
To live	Self-maintaining being different from the environment.
Death	End of self-maintained difference from the environment.
Need satisfaction	Acquiring and executing the necessities (food and energy) for life (self-maintaining being different from the environment).
Agent	“An autonomous organization that adaptively regulates its coupling with its environment and contributes to sustaining itself as a consequence.” (Barandiaran et al., 2009, p. 1).
Behavior	Agent-initiated and context-appropriate activities with expected future utility that counteract life’s precariousness and maximize agent and habitat viability.
A need	Something that, when satisfied, protects or increases agent viability.
Viability	Probabilistic distance from death (i.e., discontinued agency).
Agent viability	Agent probabilistic distance to death. To persist, all life needs to optimize viability.
Threat	A perceived reduction of context-appropriate behavioral options to include only those that allow the agent to survive.
Agency	The ability, or a measure of the ability, to self-maintain viability (through need satisfaction) for survival and thriving.
Cognition	The ability to select behavior in the service of the agent’s continued existence and flourishing.
Coping and co-creation	Two complementary forms of cognition. Coping is in the service of continued existence and flourishing in the service of flourishing. Successful coping leads to the discontinuation of its activation and promotes co-creation. (These two forms of cognition are opposed in Table 2).
Stigmergy	Building on the constructive traces that past behaviors have left in the environment (increasing habitat viability).
Authority	Expressing stigmergy.
Habitat	The environment from which agents can derive all they need to survive (and thrive) and to which they contribute to ensure long-term viability (of self and others). Note that we use the term “habitat” to include other agents, but to exclude the agent. Hence, we can speak of agent + habitat to refer to the whole of existence relevant to the agent.
Habitat viability	A measure of the degree to which the habitat can satisfy the conditions for agentic existence (i.e., satisfies its needs).
Biosphere	The sum total of all agentic traces left in the environment. Since the biosphere grew from fragile and small, to robust and extensive, we can conclude that life is a net constructive force and co-creation has been dominant.
Carrying capacity	A measure of the sum total of the life activities that a habitat can sustain.
Original perspective	A perspective on the world originating as the yet-undeveloped ability to separate individual viability from the combined viability of self and habitat, which allowed primitive life to optimize the whole, while addressing selfish needs and creating the conditions for more agentic life.
Well-being	The process of co-creation leading to high-viability agents, increased habitat viability, and long-term protection of the conditions on which existence depends. Note that this is a process, not a state or the evaluation of a state.
Motivation	Being ready to respond in a context appropriate manner.
Behavioral repertoire	The set of all context appropriate behaviors the agent has access to. Appraisal activates context-appropriate subsets of the repertoire.
Learning	The process to extend the behavioral repertoire and tune the effectivity of individual behaviors to the context
Worldview	The set of all that an agent takes as reliable (true) enough to base behavior on
Appraisal	A worldview-based motivational response to the perceived viability consequences of the present that activates context appropriate behavioral options
Core affect	Mood-level action readiness based on the appraisal of indicators of (un)safety and situationally appropriate activation of behaviors, expressed as motivations to avoid or end (coping) or motivations to perpetuate or to aim for (co-creation)
Resilience	“[T]he capacity of a system to absorb disturbance and reorganize while undergoing change so as to still retain essentially the same function, structure, identity, and feedbacks” (Walker et al., 2004)

**Table 2:** Ontologies of survival and thriving

	Ontology of survival (coping)	Ontology of thriving (co-creation)	
Languishing	Low-viability state as the outcome of a pattern of ineffective or misguided behaviors.	High-viability state as the outcome of a pattern of broadly effective behaviors.	Flourishing
Threat: behavioral constraints	Agent appraisal of viability threats, entailing a *reduction* of the set of context-appropriate behavioral options to include only those that allow the agent to survive.	Agent appraisal of the *absence* of viability threats, allowing self-guided exploration of opportunities that *enlarge* the set of context-appropriate behavioral options.	Safety: behavioral freedom
Problem	A perceived threat to agent viability that activates a pressing need and hence motivates reactive behavior.	A perceived possibility to improve (agent or habitat) viability and hence motivate proactive behavior and the expression of novel behaviors.	Opportunity
Coping	The reactive fallback mode of behavior aimed at protecting agent viability by *ending* problem states. Quick and effective deactivation of coping is the measure of success of the coping mode.	The proactive default mode of behavior aimed at producing indirect viability benefits through habitat contributions that improve the conditions for future agentic existence.	Co-creation
Reactive behavior	Behavior in response to perceived threats to viability.	Behavior aimed at setting up or protecting the conditions for co-creation.	Proactive behavior
Coping trap (Coping failure)	The continual or predominant activation of the coping mode of behavior through ineffective or counterproductive problem-solving strategies.	Prolonged or near-continual activation of co-creation.	Successful co-creation
Targeted optimization	Goal-oriented behaviors such as problem solving and task execution.	Optimizing the whole of agentic existence, while addressing selfish needs and creating ever better conditions for agentic life.	Pervasive optimization
Social mimicry	The adoption of behaviors of effective, healthy, or otherwise attractive agents leading to sameness and oneness.	Skilled contribution of self-deciding individuals who adapt and use opportunities to promote habitat flourishing.	Responsible autonomy
Learning to become less ineffective	Mimicry-based learning, where behaviors of effective, healthy, or otherwise attractive agents are copied and expressed and hence manifest shared knowledge.	The adoption of new behaviors via interactive engagement with different environments. Manifested as tacit knowledge	Learning as extending the behavioral repertoire
Main mode of cognition: intelligence	The ability to solve problems and fulfill goal-oriented tasks (to end states of pressing need).	The ability to avoid problems and co-create. (Also: The balancing skills to contribute to the biosphere).	Main mode of cognition: generalized wisdom
Inadequacy	The tendency to self-create, prolong, or worsen problems that keep on activating the coping mode. An inadequate agent is predominantly coping, but unsuccessful in ending the activators of coping.	The skill to avoid problems or end them quickly so that coping is rare and co-creation prevalent. An adequate agent is a predominant co-creator.	Adequacy
Coping adequacy	The skill to solve pressing problems (ending the need to cope) or to mitigate their impact through control of the environment and constraining agency (continuing coping).	The skill to avoid and end problems through harmonizing relations, (inter-agent) conflict mitigation, and promoting unconstrained innate behaviors.	Co-creation adequacy
In-group	A group of individuals sharing similar limits on adequacy (and worldview).	A group of individuals who each freely and self-guidedly contributes whatever benefit their adequacy offers.	Community
Out-group	Individuals who violate sameness and oneness and hence frustrate coordinated coping.		
Security	A situation or state where viability threats-to-self are brought under control.	A situation or state with positive indicators of the absence of viability threats.	Safety
Power	The ability to realize intended outcomes by effortfully shaping and controlling the habitat and the activities of the agents that comprise it. Exercising power is a way to be authoritative.	Effortless action expressing authority through harmonizing a diversity of agentic interests by promoting natural agentic dynamics and development.	Wu Wei

## Section 1 — Core Cognition

Is well-being unique to humans, or animals, or does it pertain to life in general? We argue that well-being is a foundational concept that can best be understood as pertaining to all living entities, through a shared motivation for survival and thriving. In this section we define a set of key terms defining **core cognition** (Andringa, van den Bosch, & Wijermans, 2015): the foundational cognition shared by all life to secure its continued existence and flourishing. In Part 2, we show that core cognition allows us to unify a number of well-known — but still unconnected — phenomena in psychology, such as the structure of identity and how the concepts of security and safety lead, respectively, to states of pathological normality or healthy personal and interpersonal development.

### Being by Doing

A living entity is different from a dead entity because it self-maintains this difference. **To live** entails self-maintaining and self-constructing a “far from equilibrium state”. The work of Prigogine (1973) showed that, for thermodynamic reasons, such an inherently unstable system can only be maintained via a continual throughput of matter and energy (e.g., food and oxygen). **Death** coincides with the moment self-maintenance stops. From this moment on, the formerly living entity moves towards equilibrium and becomes an integral and eventually indistinguishable part of the environment.

A living entity “is” — exists — because it “does”: it **satisfies its needs** by maintaining the throughput of matter and energy by “*adaptively regulating its coupling with its environment so that it sustains itself*” (Andringa et al., 2015; Barandiaran, Di Paolo, & Rohde, 2009 p. 8). An autonomous organization that does this is called a “living agent” or an **agent** for short (Barandiaran et al., 2009). Note that we refer to an agent when the text pertains to life in general and is part of core cognition. Where we specifically refer to humans we use the term “person”. The term “individual” can refer to both, depending on context.

Life is precarious (Di Paolo, 2009), in the sense that it must be maintained actively in a world that is often not conducive to self-maintenance and where both action and inaction can have high viability consequences (including death). We refer to **behavior** as agent-initiated context-appropriate activities with expected future utility that counteract this precariousness and minimize the probability of death. Behavior is always aimed at remaining as viable as possible, since harm — viability reduction — can more easily end a low-viability than a high-viability existence.

A pattern of behaviors that effectively optimizes viability leads to **flourishing**, while a pattern of ineffective or misguided behaviors leads first to **languishing** and eventually to death. Life is “being by doing” the right things (Froese & Ziemke 2009, p. 473). **Viability** is a holistic measure of the success or failure of “doing the right things”, since it is defined as the probabilistic distance from death: the higher the agent’s **viability**, the lower the probability of the discontinuation of life. A walrus that falls offa cliff may be perfectly healthy, but it has zero viability, since it will die the moment it hits the ground. While healthy, it is in mortal and inescapable danger, and hence unviable. In general, **threat** signifies a perceived reduction of context-appropriate behavioral options that allow the agent to survive. Maximizing viability (flourishing) and minimizing danger (survival) constitute basic motivations of life. In fact, we call any system **cognitive** when its behavior is governed by the norms of the system’s own continued existence and flourishing (Di Paolo & Thompson, 2014). This is also a reformulation of “being by doing”.

### Cognition for Survival and Thriving

**Agency** entails **cognition**: behavior selection for survival (avoiding death) and thriving (Barandiaran et al., 2009) (optimizing viability of self and habitat). We have argued that cognition for survival is quite different from cognition for thriving (Andrin ga et al., 2015). Cognition for survival is aimed at solving problems, where a **problem** is any perceived threat to agent viability, interpreted as a pressing need that activates **reactive behavior.** We called this form of cognition **coping.** In humans, (fluid) intelligence is a measure of problem-solving and task-completion capacity and manifests coping. The objective of coping is ending/solving the problems that activated the coping mode, so ideally coping is a temporary state. We refer to the problem-solving *ability,* including successful test and task completion ability (Gottfredson, 1997; van der Maas, Kan, & Borsboom, 2014), as **intelligence.**

However, when the agent’s problem solving is inadequate and problems are not solved and are potentially worsened or increased, the perceived viability threat remains activated and the agent is trapped in the coping mode of behavior. A **coping trap** keeps the agent in continued threatened viability, and hence in behaviors aimed at short-term self-protection in suboptimal states that are far from flourishing. Maslow (1968) calls this deficiency (D) cognition, since it is ultimately activated by unfulfilled needs. It is a sign that the intelligence of the agent failed to end (solve) problem states.

While the coping mode of behavior is for survival, the co-creation mode is for flourishing. Successful coping leads to solved problems and satisfied needs, and hence to its deactivation. Therefore, co-creation is the default mode of cognition and coping is — ideally — only a temporary fallback to deal with a problematic situation. Continued activation is the success measure of the co-creation mode and avoiding problems (or dealing with them before they become pressing) is, therefore, the main objective of co-creation. It is essentially proactive behavior (thus not just “proactive coping”, since successful coping leads to its deactivation). Maslow (1968) refers to co-creation as being (B) cognition, and we described it as pervasive optimization and “generalized wisdom”, for reasons which will become apparent. The objective of co-creation is pro-actively producing indirect viability benefits through self-guided habitat contributions that improve the conditions for future agentic existence.

This is known as **stigmergy**: building on the constructive traces of past behaviors left in the environment (Doyle & Marsh, 2013; Gloag et al., 2013; Heylighen, 2016b; 2016a) and that, in the aggregate, gradually increase habitat viability. This expresses **authority** as a shaping force in the habitat (Marsh & Onof, 2008), via influencing others through habitat contributions. **Habitat** is defined as the environment from which agents can derive all they need to survive (and thrive) and to which they contribute to ensure long-term viability of the self and others.

**Habitat viability** is a measure of the potential of the habitat to satisfy the conditions for agentic existence (i.e., satisfied agentic needs). For example, a habitat can be deficient in the sense that its inhabitants continually have unfulfilled needs (and hence are in the coping mode). The habitat can also be rich, so that pressing needs can easily be satisfied and co-creative contributions can perpetuate and enhance habitat viability.

The **biosphere** grew from fragile and localized to robust and extensive, so we know beyond doubt that life on Earth is, in the aggregate, a constructive force. It is the co-creation mode’s contributions to habitat viability that explain this. In fact, the biosphere can be seen as the outcome of stigmergy: the sum total of all agentic traces left in the environment since the origin of life (Andringa et al., 2015). Co-creation and generalized wisdom as the main cognitive ability drive the biosphere’s growth and gradually increase its **carrying capacity:** the sum total of all life activity in the biosphere. This makes co-creation the most authoritative influence on Earth. Coping is also an important authoritative influence, but it is limited to setting up and maintaining the conditions for pressing need satisfaction.

**Figure 1.**
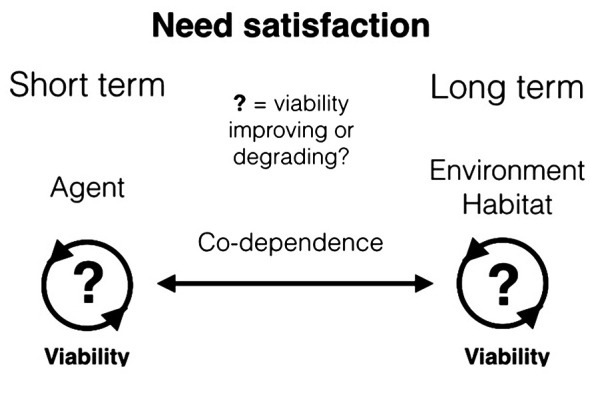
Life’s demand: maintaining and increasing viability of self and habitat (based on Andringa & Angyal, 2019). Pervasive optimization of agent and habitat viability leads to increased carrying capacity and more life.

*Figure 1* presents the co-dependence of acting agents on their habitat. The habitat comprises the aggregate of agentic activities, but is not an actor itself. Hence, a viable habitat is composed of the sum total of previous co-creative agentic traces that form a resource to satisfy the conditions on which current agentic existence depends. This entails that, signified by the question marks, agents should be aware not only of their own viability, but also of habitat viability. In fact, we have argued (Andringa et al., 2015) that early, primitive life forms were not yet able to separate the self from the co-dependence of self and habitat. This leads to an **original perspective** on the combined viability of agent and habitat, which allowed their primitive cognition to optimize the whole, while addressing selfish needs and co-create ever better conditions for agentic life. This can be termed **pervasive optimization** and it expresses an emergent purpose of life on Earth to produce more life. Albert Schweitzer (1998) formulated a slightly weaker version of this: “I am life that wills to live in the midst of life that wills to live.”

### Well-Being and Adequacy

Pervasive optimization is the driver of **well-being.** We propose that successful wellbeing, with a focus on “being” and hence interpreted as a verb, can best be understood as a co-creation process leading to high-viability agents, increased habitat viability, and long-term protection and extension of the conditions on which existence depends.

The two modes of behavior have quite different impacts on the habitat and, by extension, the biosphere. The coping mode is aimed at protecting and improving agent viability with whatever means the agent has access to. Since the objective is avoiding death, the motivation is high, which entails that habitat resources can be sacrificed for self-preservation purposes. **Inadequacy** can be defined as the tendency to self-create, prolong, or worsen problems that keep an agent in the coping mode. When a habitat is dominated by inadequate agents, as is characteristic of a social level coping trap, habitat viability cannot be maintained, let alone increased. From the perspective of coping, life is at best a zero-sum game.

Alternatively, **adequacy** can be defined as the ability to avoid problems or end them quickly so that coping is effective and rare. Now co-creation is prevalent so that habitat viability is protected, carrying capacity increases, and long-term need satisfaction is secured. Co-creation is, as the term suggests, a *more than zero-sum game*. This is, as argued above, the true basis of well-being. Due to its lack of “co-creation”, coping *protects* lower levels of well-being and, at best, resolves (or otherwise takes care of) viability threats (in the sense of removing symptoms of low well-being), while co-creation allows both agent and habitat flourishing.

The inadequacy/adequacy dimension might underlie the proposed single dimension of psychopathology termed p (Caspi & Moffit, 2018; Lahey et al., 2012). This has been conceptualized as “a continuum between adaptive and maladaptive functioning”, “successful versus unsuccessful functioning”, a disposition for negative emotionality or impulsive responsivity to emotion, and unrealistic thoughts that manifest in extreme cases as delusions and hallucinations (Smith et al., 2020). All descriptions fit with our interpretation of inadequacy as the tendency to self-create, prolong, or worsen problems, and adequacy as the ability to avoid problems or end them quickly.

Welzel and Inglehart (2010) argue, from the perspective of cultural evolution, that “feelings of agency are linked to human well-being through a sequence of adaptive mechanisms that promote human development, once existential conditions become permissive”, which is a formulation of the dynamics of *Figure 1*. They argue that “greater agency involves higher adaptability because for individuals as well as societies, agency means the power to act purposely to their advantage”. This uses the concept of agency as a *measure* of the ability to self-maintain viability, which is related to adequacy.

### Behavioral Repertoire and Worldview

Living agents, per definition, need to express behavior to perpetuate their existence. And with every intentional action, the agent implicitly relies on the set of all that it takes as reliable enough (i.e., true enough in the sense of reflecting reality as it is) to base behavior on. We refer to this set as the agent’s **worldview.** A worldview should be a stable basis, as well as developing over time because it is informed by the individual’s learning history. An agent’s worldview informs its appraisal of the immediate environment. This may be an appraisal of its viability state: whether the habitat is safe or not, or whether it judges the current situation as manageable, too complex, or opportunity filled.

These are basic appraisals shared by all of life that seem to be reflected in the psychological concept of core affect (Russell, 2003). Core affect is a mood-level construct that combines the axis unpleasurable/pleasurable with an arousal axis spanning deactivated to maximally activated. Core affect is intimately and bidirectionally linked to appraisal (Kuppens, Champagne, & Tuerlinckx, 2012; van den Bosch, Welch, & Andringa, 2018); and refers directly to whether one is free to act or forced to respond: whether one can co-create proactively or has to cope reactively. Hence **appraisal** is a worldview-based motivational response to the perceived viability consequences of the present state of the world. It is motivational, but not yet action. As such, appraisal resembles Frijda’s (1986) emotion definition as “action readiness”. Which fits with the notion that all cognition is essentially anticipatory:

Cognitive systems anticipate future events when selecting actions, they subsequently learn from what actually happens when they do act, and thereby they modify subsequent expectations and, in the process, they change how the world is perceived and what actions are possible. Cognitive systems do all of this autonomously. (Vernon, 2010, p. 89)

The anticipation of the development of the world (comprised of self and environment) refers back to what we earlier introduced as the “original perspective” on the combined viability of agent and habitat, which allowed the first life forms to optimize the whole, while addressing selfish needs and creating ever better conditions for more agentic life. Core affect is a term adopted from psychology (Russell, 2003), which we here generalize to all of life. Core affect is a relation to the world as a whole and not a relation to something specific in that world. Like moods, core affect does not have (or need) the intentionality (directedness) of emotions and it is, unlike emotions, continually present to self-report (van den Bosch et al., 2018).

**Figure 2.**
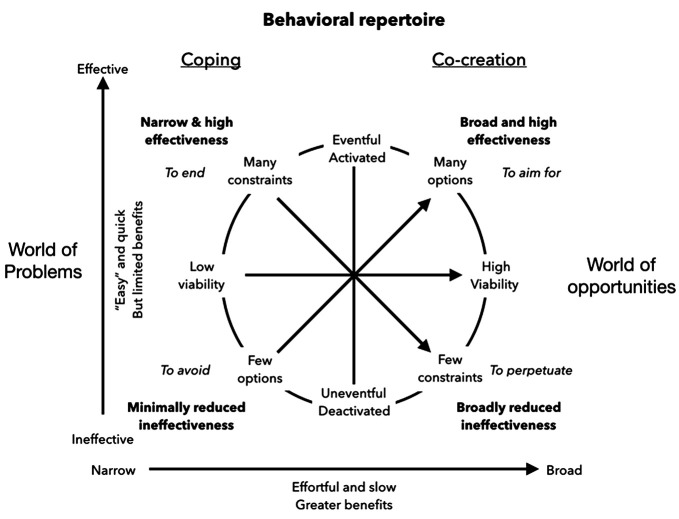
Behavioral repertoire. The concepts around the circle refer to appraisal and the verbs in italic to basic motivations. The descriptions in bold and the outer axes refer to the structure of behavioral (in)effectiveness.

The human worldview is, of course, filled with explicit and shared beliefs, opinions, facts, and ideas interpreted with and filtered by experiential knowledge. This worldview informs whether a situation is appraised as dangerous (whether avoidance or approach is appropriate). This holds also for a general agent: when the agent judges the situation as safe, it can express unconstrained natural behaviors, since it has to satisfy few constraints. If the situation is safe and opportunity-filled, the agent can be interested and learn, but if the situation imposes many constraints, the agent tries to end these by establishing control. And in a deficient environment the agent is devoid of opportunities (which in humans may correspond to boredom or, in case of lost opportunities, to sadness). Core affect then is expressed as motivations to avoid or end (coping) or motivations to perpetuate or to aim for (co-creation). We have depicted this in *Figure 2*.

Appraisal of reality refers to the behavioral consequences of the current state of the world and it is a form of basic meaning-giving that activates a subset of context appropriate behavioral options (van den Bosch et al., 2018). This leads to **motivation** as being ready to respond to the context appropriately. We define the set of all possible behaviors — appraisal- and worldview-dependent — as the **behavioral repertoire.** The richer the behavioral repertoire, the more diverse context-appropriate behaviors the agent can exhibit. The more effective its behavioral repertoire, the more effective the agent becomes in realizing intended outcomes and the more adequate the agent is. Conversely, the less effective the context-activated behaviors, the more inadequate the agent is. **Learning** either reduces the ineffectiveness of behaviors or it expands the behavioral repertoire.

**Expanding the repertoire** results from an individual discovery path through a representative sample of different environments and participative learning opportunities. Broadening is effortful and potentially risky, but ultimately rewarding. Fredrickson’s (2005) “broaden-and-build” theory fits here by proposing that positive emotions — indicating the absence of problems and hence co-creation — help to *extend* the scope of behavioral options. This type of learning leads to individual skills that are, through the individual discovery path, difficult to share. This is manifest in humans as implicit or tacit knowledge (Patterson, Pierce, Bell, & Klein, 2010) and well-developed agency.

**Reducing the *ineffectiveness* of behaviors** is essential in problematic (coping) situations. This may entail adopting, through **social mimicry**, the behaviors of (seemingly) more successful, healthy, or otherwise attractive agents. The adoption of presumed effective behaviors manifests shared knowledge. Mimicry is a quick fix and works wherever and as long as the adopted behaviors are effective. As a dominant learning strategy, mimicry leads to a coordinated situation of sameness and oneness. Coordinated agents make their adequacy conditional upon the narrow set of situations where the mimicked behaviors work. These agents may be intolerant to others who frustrate sameness and oneness. They may express this intolerance by selecting behaviors that enforce social mimicry on non-mimickers. The more they feel threatened, the more they feel an urge to restore the conditions for adequacy and the more intolerant to diversity they are. In humans this is expressed as the authoritarian dynamic (Stenner, 2005).

### Core Cognition: Key Terms

This discourse leads to a selection of core cognition’s key concepts and their definition, which is presented in *Table 1*.

## Section 2 — Coping and Co-Creation

This section addresses the quite different and complementary features of coping and co-creation. We need both, because successful coping maximizes time for co-creation. The complementarity of the two modes, as two separate ontologies that disagree on many aspects, might be the root of life’s **resilience.** Where resilience is defined as “the capacity of a system to absorb disturbance and reorganize while undergoing change so as to still retain essentially the same function, structure, identity, and feedbacks” (Walker, Holling, Carpenter, & Kinzig, 2004). We originate resilience in the agent’s ability to anticipate and predict.

### Anticipation and Predictability

Coping and co-creation are abilities in psychology, skills, and tacit knowledge (Patterson et al., 2010) expressed as behavior in response and appropriate to how the agent appraises its habitat context. Of course, agent-initiated actions change the habitat state to which other agents may respond, which, in turn, changes the habitat state. Since the habitat may change even without direct agentic influences, agents exist in an evolving world in which they must position themselves to protect and enhance self and habitat viability. To exist in such an environment, the agent needs anticipatory models (Vernon, 2010) of the state of the self and the habitat. It must update these actively, and choose its behavior to realize benefits to the self and the habitat. In this open environment, even the best agent-generated model leads only to partial predictability. Coping and co-creation strategies increase partial predictability, but use different strategies and complementary logics.

### Coping

Coping makes the world more predictable by reducing its complexity and creating systems (of agents or objects) with more predictable behavior, which bring threats-to-self under control — which requires energy, resources, and continual maintenance — and promote **security.** The coping mode’s goal is to end perceived viability threats, and coping success entails the discontinued need for its activation. Hence, it is goal-oriented (like problem solving and task execution) and endowed with a sense of urgency to avoid (further) viability deterioration that justifies the exploitation of previously created viability. Any deviation from manageable order — unfamiliar events or deviant agent behavior — is seen as an unwanted intrusion to be counteracted. Hence, coping leads to an effortfully controlled environment that minimizes unpredictability and diversity. If the threat level — i.e., the expected negative viability impact — increases, so does the drive to suppress diversity.

Since coping is goal-oriented and intends to reduce complexity, it favors shared rules (in general, shared knowledge) and behavioral mimicry. The more agents follow the same rules with great precision, the more predictable agents and the habitat become. Coping promotes the spread and precise execution of a single set of behavioral rules, and endorses an urge to correct or suppress any unwanted diversity. This is a form of social mimicry (Chartrand & van Baaren, 2009), which might not only lead to the spread of effective behavior, but also to a *“degree of entanglement”* (Combs & Kribner 2008, p. 264), emergent collective behavior (via mimicry or rules), and a group-level perspective.

In human societies, bureaucracy, the military, large corporations, and strict manifestations of religions and ideologies are examples of coping logic. Technology, from the very primitive to complex, like computers, depicts the best coping by producing precise outputs, as long as the physical environment (the tool and its necessary resources) and the user operate within very tight constraints; this entails trained behaviors.

Coordinated agentic behavior, such as social mimicry, is endorsed by agents who expect benefits from more sameness and oneness. Agents with similar needs share similar coordination benefits, but that is unlikely for agents with different needs or those with other (even potentially better) strategies. In fact, imposed external coordination might be detrimental. Differences in expected benefits lead to a separation into in-groups and out-groups. An **in-group** is a group of agents who express a degree of oneness and sameness through social mimicry and hence share adequacy limits, perceptions of what is beneficial, how to realize these benefits, and what endangers the realization of these benefits. **Out-groups** do not share these limits, either because they have other limits or because they are less limited. By violating sameness and oneness, out-groups frustrate coordinated coping in the eyes of in-groups. Note that out-groups might not even know they are assigned to the out-group and might not raise their defenses.

In-groups (as a manifestation of coping) see the risk of frustrated coordinated behavior as an existential threat, which justifies exploiting or suppressing out-groups and the habitat alike. Habitat and out-group exploitation may activate out-group resistance that makes goal achievement more difficult. So, the better the in-group is able to control out-groups and habitat, the more likely they are to realize the intended results. Due to its problem-solving nature, coping manifests “*the ability to realize intended outcomes”*, which is Bertrand Russell’s (1938) definition of **power.** Hence coping behaviors are a manifestation of power generalized to generic agents.

The coping mode’s manifestation of authority is typically power based, in the sense that it sets up habitat conditions for reduced diversity, increased predictability of agent behavior to facilitate intended outcomes and to bring viability threats-to-self under control (**security**). This is known as coercive authority (as opposed to legitimate authority (Hofmann, Hartl, Gangl, Hartner-Tiefenthaler, & Kirchler, 2017). Coercive power generally (but not necessarily) leads to benefits for the in-group to the detriment of out-groups and the wider habitat: the zero-sum game that in humanity is associated with manifestations of authoritarianism (Stenner, 2005) and the tragedy of the commons (Hardin, 1968).

### Co-creation

Co-creation does not reduce complexity; instead, it makes the world more predictable by promoting unconstrained natural behavior and easy need satisfaction through promoting and communicating efforts that facilitate and maintain habitat viability. This creates a safe environment where **safety** is defined as “a situation or state with *positive indicators* of the absence of viability threats” (van den Bosch et al., 2018). This communicated absence of threats is a logical necessity, since absence can otherwise not be established. The positive indicators of safety — signs of unforced agentic behavior — allow agents in the habitat to co-create without having to be on alert for (unexpected) danger. This allows the uninterrupted functioning of a self-organizing network of interacting agents that satisfy needs most naturally, while minimizing negative impacts and promoting coexistence and even collaboration. Human friendships depend on this logic, and they have, like all co-creation processes, no stable outcome or goal other than providing a safe context for growth and flourishing.

This is the complement of coordinating other agents’ behavior (which characterizes coping). Unconstrained natural behavior does not need guidance, since the agents do whatever comes naturally and return to this when constraints are lifted. This harmony between what is possible and what comes naturally stabilizes the habitat, leads to more communicated safety, and increases predictability through the reduction of interagent tension, which otherwise might activate coping as a fallback. Co-creating agents should become aware of the needs of others and what comes naturally to themselves, others with similar needs, others with different needs, and the wider habitat’s dynamics. They have to optimize it all in the context of everything else and over all timescales (we referred to this as “pervasive optimization”, Andringa et al., 2015), which is a direct reference to Sternberg’s definition of wisdom:

The application of tacit knowledge towards the application of a common good through a balance among intra-, inter-, and extra- personal interests to achieve a balance among adaptation to existing environments, shaping of existing environments, and a selection of new environments, over the long term as well as the short term. (Sternberg, 1998)

This definition is somewhat human-centered and can easily be generalized to all life, all agentic interests, all habitats, and all time-scales. And since tacit knowledge refers to skills, Sternberg’s definition can be generalized to “the balancing skills to contribute to the biosphere”. This is what we refer to as **generalized wisdom.**

Whereas the application of power generally (but not necessarily) produces benefits to an in-group at the detriment of out-groups, proper co-creation leads to broadly constructive benefits and is a more than a zero-sum game. As we argued, this has driven and arguably still drives biospheric growth. Note that many agents might still suffer; co-creation manifests broad net benefits, not the absence of harm or suffering. Typically co-creating agents form a **community,** a group of individuals who each freely and self-guidedly contributes whatever benefits their adequacy can bring.

Co-creating agents need to act on what comes naturally to agents and habitats. They must learn how to promote more natural behavior and prevent behavior leading to broadly detrimental consequences. The Daoist key term **Wu Wei**, reflects this, since it “*means something like ‘act naturally,’ ‘effortless action,’ or ‘nonwillful action’* ” (Littlejohn, 2003). Characteristically, it completely misses the urgency of coping strategies and the effort associated with exercising power. *Wu Wei* is also a way to be authoritative:

... individuals emerge authoritative and powerful as part and parcel of an interconnected web of forces. Therefore, a crucial back-and-forth tug between the self and the various influences and authorities surrounding it is woven in the very fabric of what it means to be a fully attained and empowered individual. (Brindley, 2010, pp. xxvii–xxviii)

*Wu Wei* is a quite different conception of authority, since it does not pertain to realizing specific intended results, but instead is aimed at pervasive optimization (Andringa et al., 2015) and becoming “a fully attained and empowered individual” as “part and parcel of an interconnected web of forces”, or what Maslow (1954) refers to as self-actualization. It is this growth process that drives identity development (see Part 2), as much as it promotes general well-being.

Co-creation expresses and relies on highly skilled behaviors of many **responsible autonomous** individuals, who adapt to and use the possibilities of changing situations. As such it is not easy to maintain and somewhat fragile; the highest co-creative quality is difficult to maintain and generally transitory. This is quite different for coping, which relies on more basic strategies such as mimicry and rule-following, and which can be both stable and stultifying.

### Two Ontologies

The complementary properties and behavioral logic of coping and co-creation lead often to opposing strategies. Both aim to increase habitat predictability. Coping does that by imposing behavioral constraints and habitat control to counteract adequacy limits. Co-creation instead promotes the creation of a never-stable network of behaviors that come naturally and unconstrained and that distribute the responsibility for habitat viability over all contributing agents. This implicitly assumes that participants are willing and able to alleviate their adequacy limits and grow in their ability to co-create.

Coping and co-creation are both essential, but successful coping is short-lasting and effective; it ends the cause of its activation and restores co-creation as the behavioral default. Unsuccessful coping is ineffective, and hence prolonged. And since the causes for its activation remain valid, it precludes co-creation. This entails that individuals who predominantly cope or co-create develop quite different worldviews, strategies, values, and identities. Hence, they might not be able to understand one another or to collaborate effectively.

*Table 2* shows the two separate ontologies of coping and co-creation. It organizes and relates the concepts within each ontology through matching them to complementary concepts and/or roles in the other ontology. That we are able to do that on a consistent basis, suggests not only the structural complementarity of coping and co-creation, but also that we are uncovering some basic tenets of life and cognition.

We consider the selection, matching, and precise formulation of these concepts an ongoing process. Hence, its formulations will develop over time; the formulation in the table is our current best.

In Part 2 of this paper, we apply and extend the proposed framework to identity development and we apply it on a metatheoretical level to two approaches to general well-being: ontological security as a manifestation of coping, and psychological safety as a manifestation of co-creation. This leads to the extension of both tables and an improved definition of co-creation and the two ontologies that comprise it.

## Conclusion

In this paper we proposed that human psychology is rooted in core cognition, the presumed cognition shared by all of life. We used the defining properties of life to propose fundamental terms in order to describe the key features of core cognition (see *Table 1*). Many of these terms had already been defined in the context of enactive cognition, psychology, or elsewhere; but had never been combined in a single framework.

We concluded that the main demand of life is to maintain and increase the viability of self and habitat. Pervasive optimization of the co-dependence of agent and habitat is the driver of individual and collective well-being. In the aggregate, this drives/stipulates biospheric growth (see *Figure 1*). In humans, this skill manifests as wisdom.

We defined cognition as the ability to select behavior in the service of the agent’s continued existence and flourishing and we described the structure of behavioral (in) effectiveness in terms of both increasing the effectiveness and increasing the scope of the agent’s behavioral repertoire (see *Figure 2*). This naturally coupled to core affect, the appraisal of the environment, and motivations.

We derived two complementary and often contradictory ontologies of behavior: co-creation and coping. Co-creation is the default mode that aims to perpetuate itself through preventing problem states by promoting unconstrained natural behavior and easy need satisfaction. Co-creation optimizes all in the context of everything else; it is the cognition for thriving. Coping is the fallback strategy intended to solve problems quickly and urgently by reducing complexity and promoting more predictable behaviors through imposing limits on behaviors and social mimicry. It is the cognition for survival.

An inadequate agent expresses the tendency to self-create, prolong, or worsen problems that keep on activating the coping mode. An inadequate agent remains predominantly in the coping mode, as they are unsuccessful in ending the activators of coping. Conversely, an adequate agent has the skills to avoid problems or end them quickly so that coping is rare, and co-creation prevalent. We suggested that the proposed p, as a single dimension of psychopathology, reflects inadequacy.

While we constructed the ontologies of coping and co-creation (see *Table 2*) we noticed that each entry on one ontology corresponded with a matching, but intrinsically disparate entry, in the other ontology. Since it is not directly obvious why this is the case, it warrants further investigations. Overall, we consider the selection, matching, and precise formulation of these concepts an ongoing process. Hence, conceptual formulations will develop over time; the formulation in the tables is our current best.
